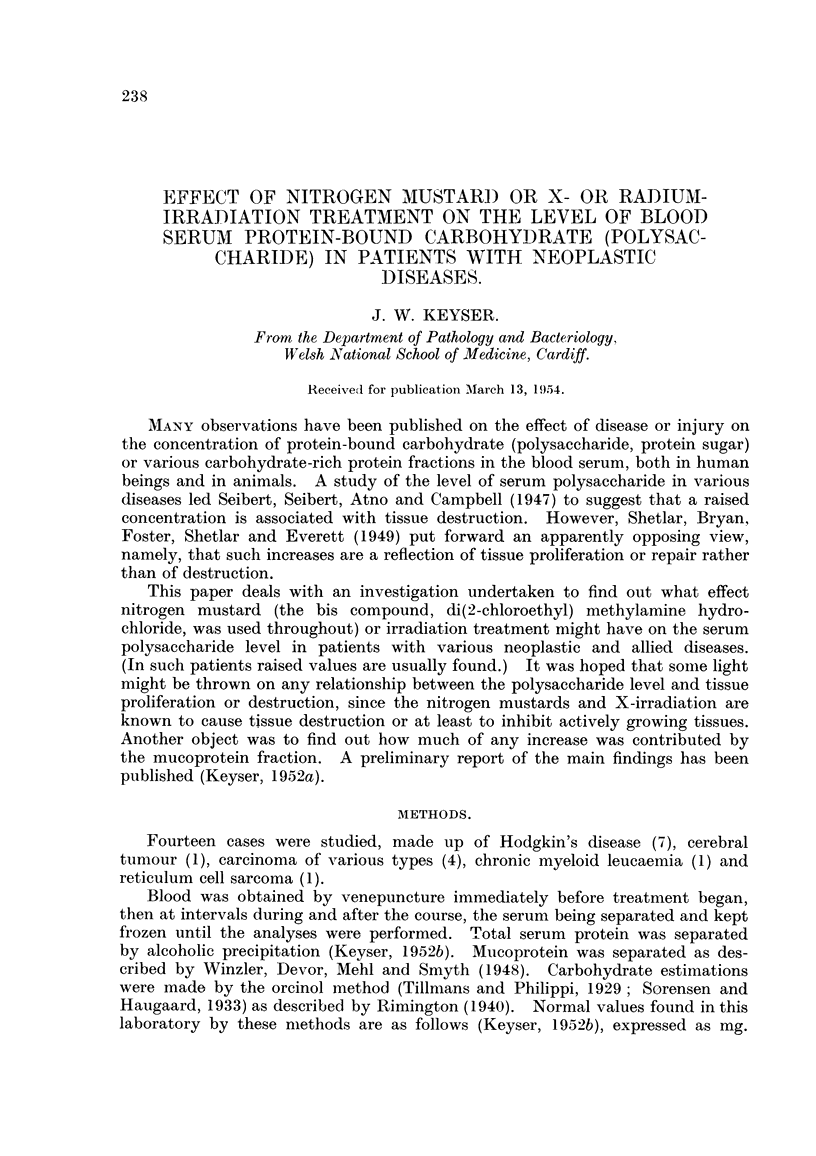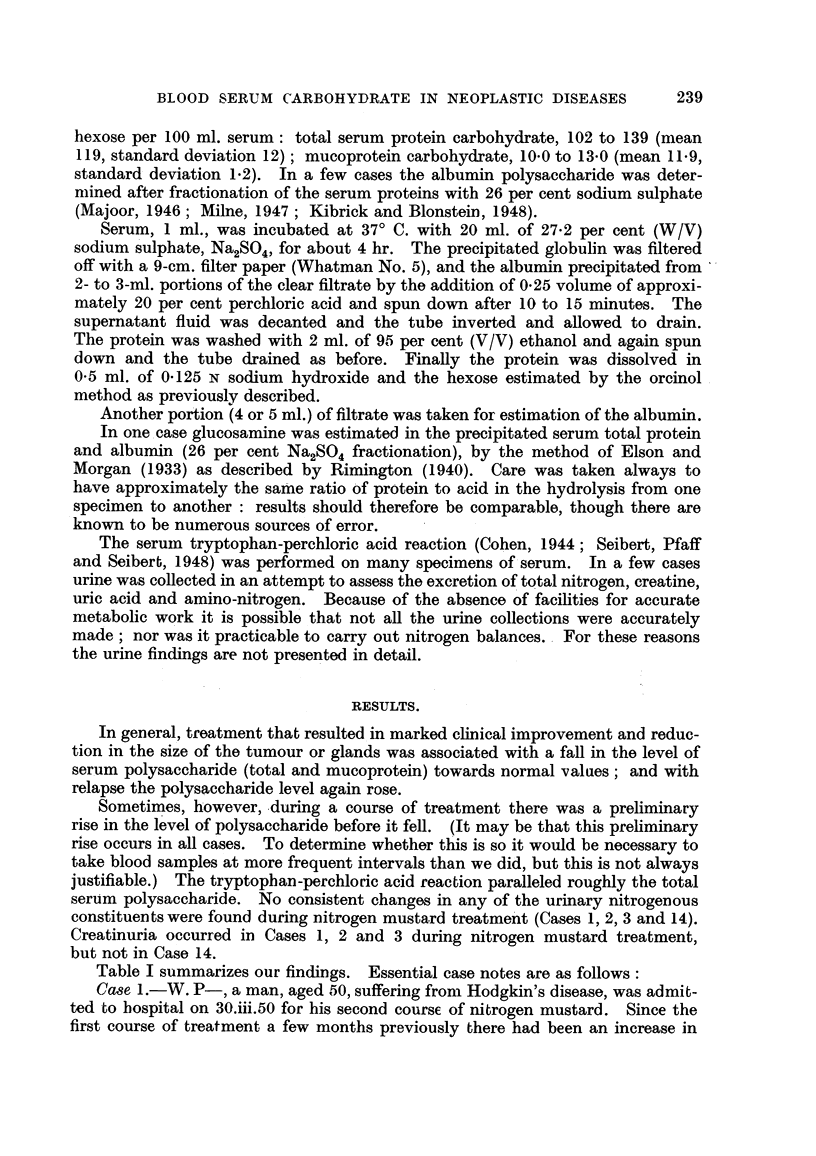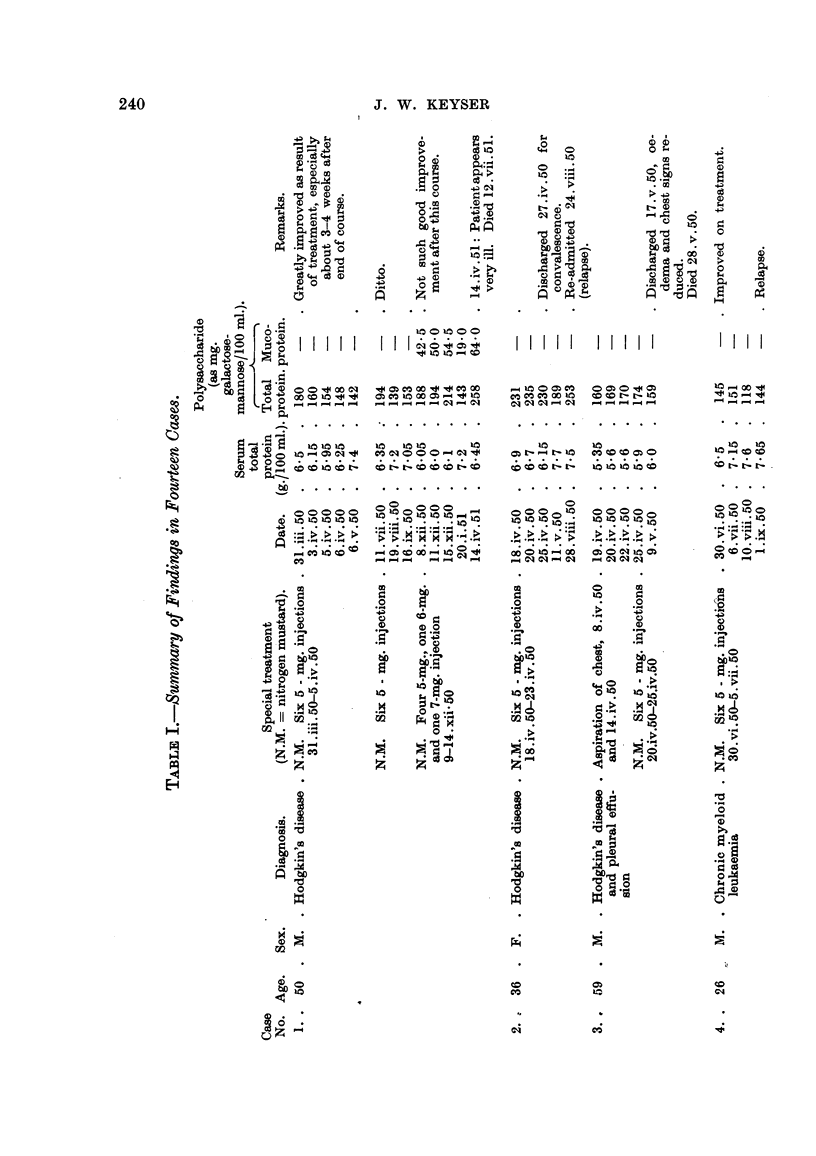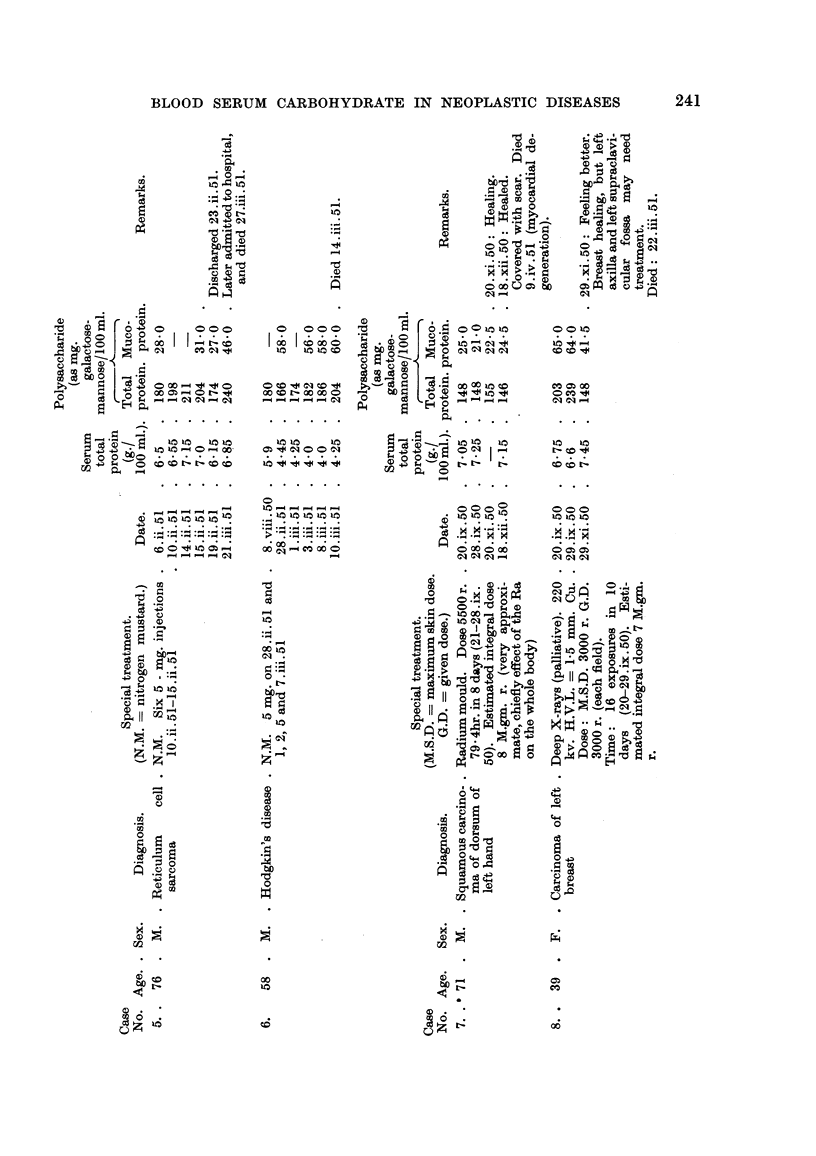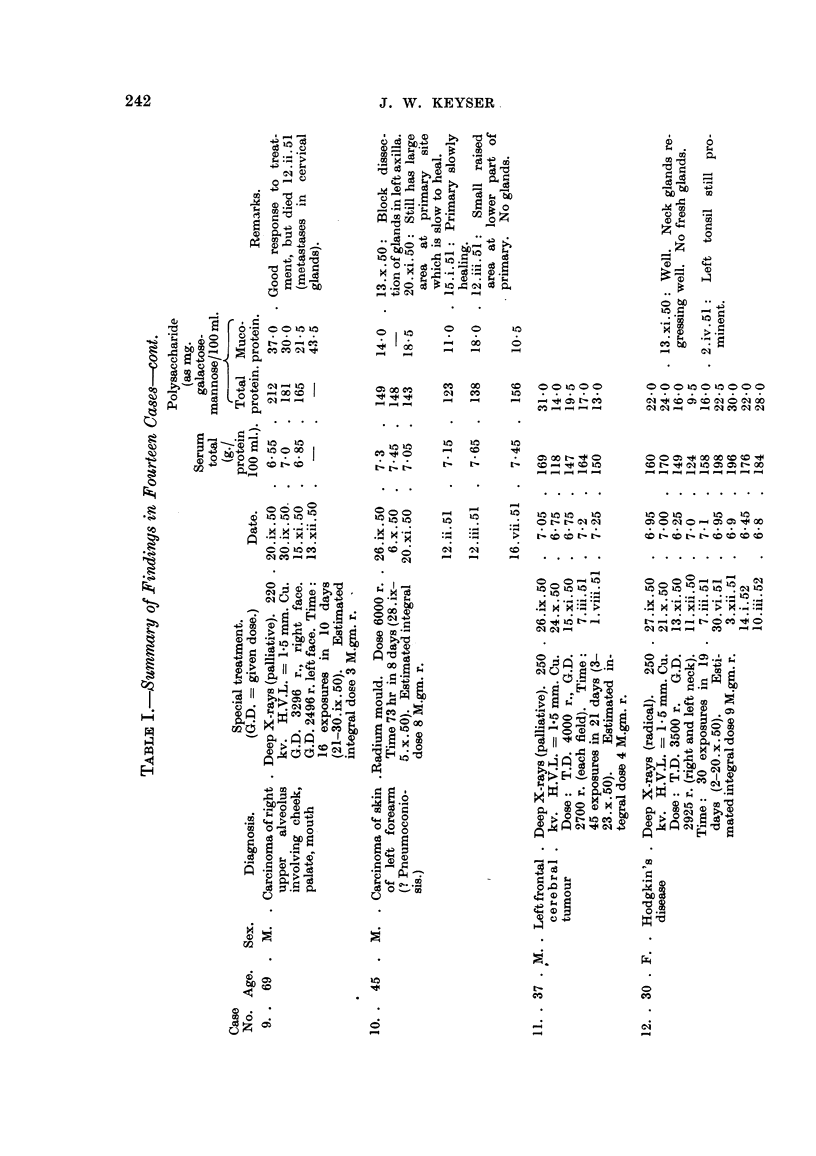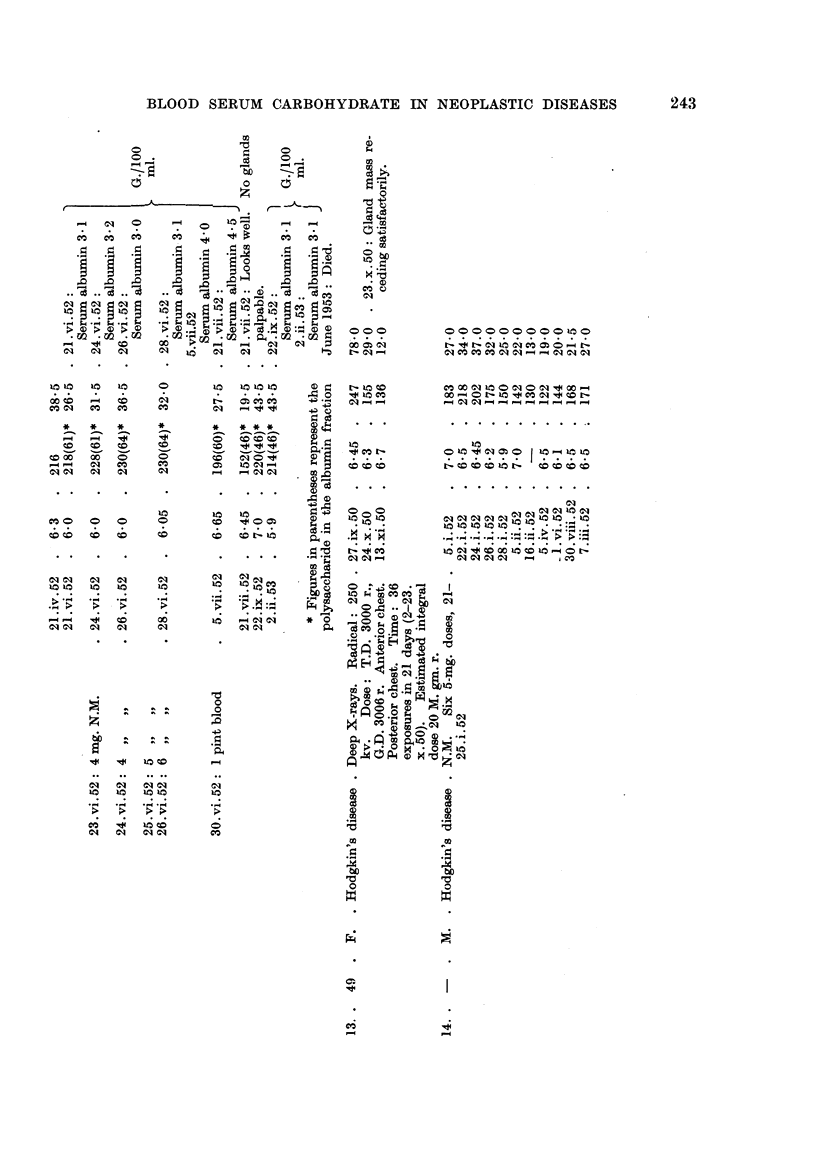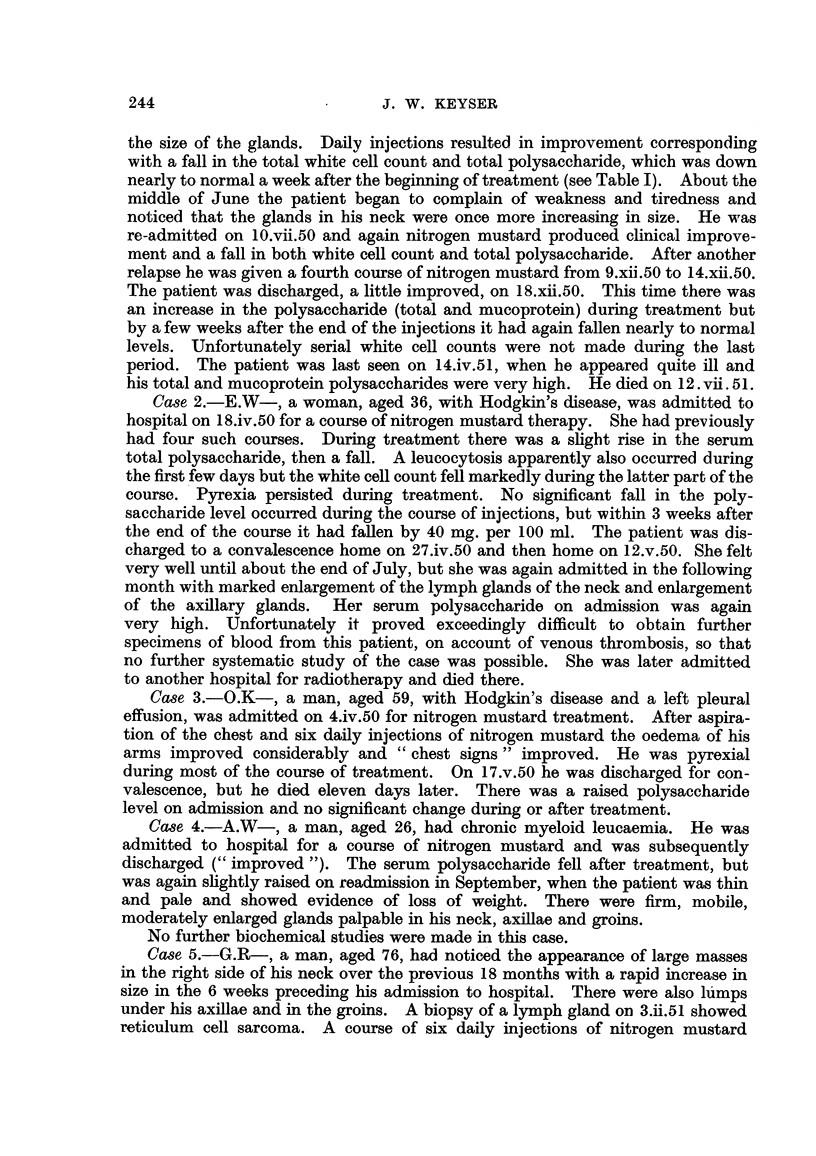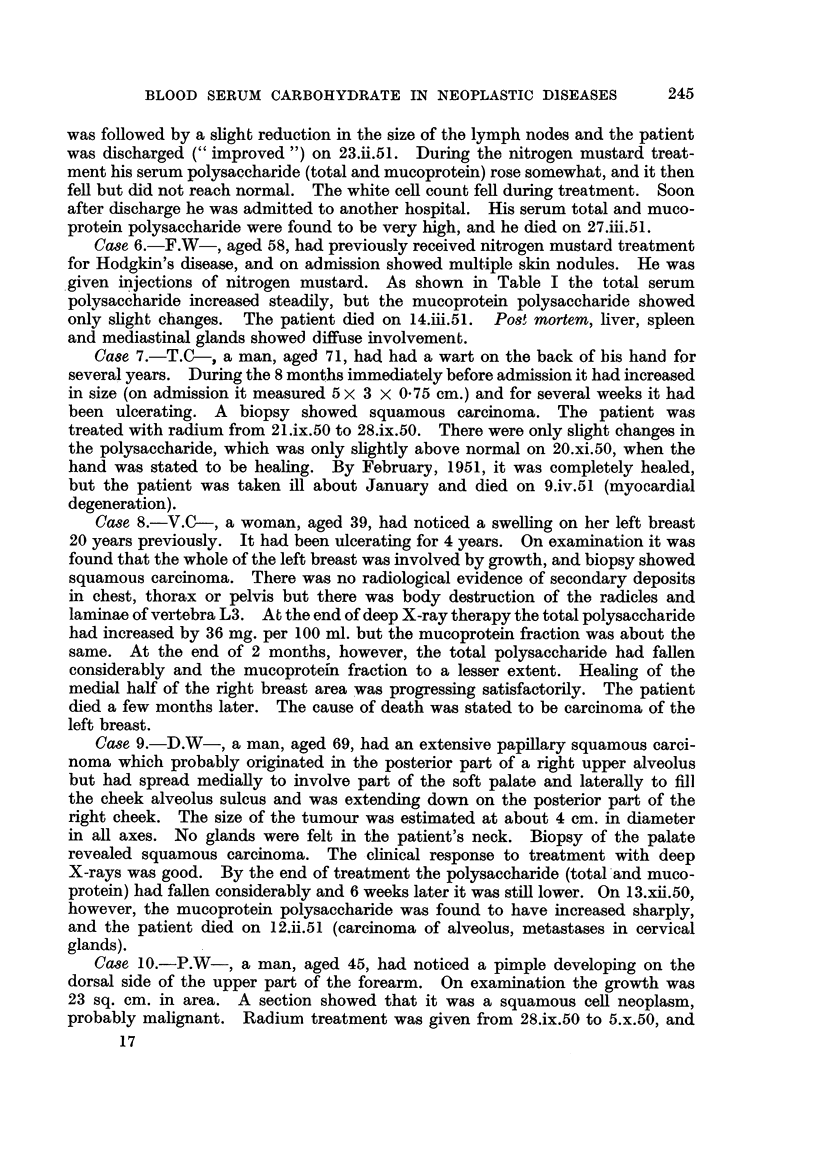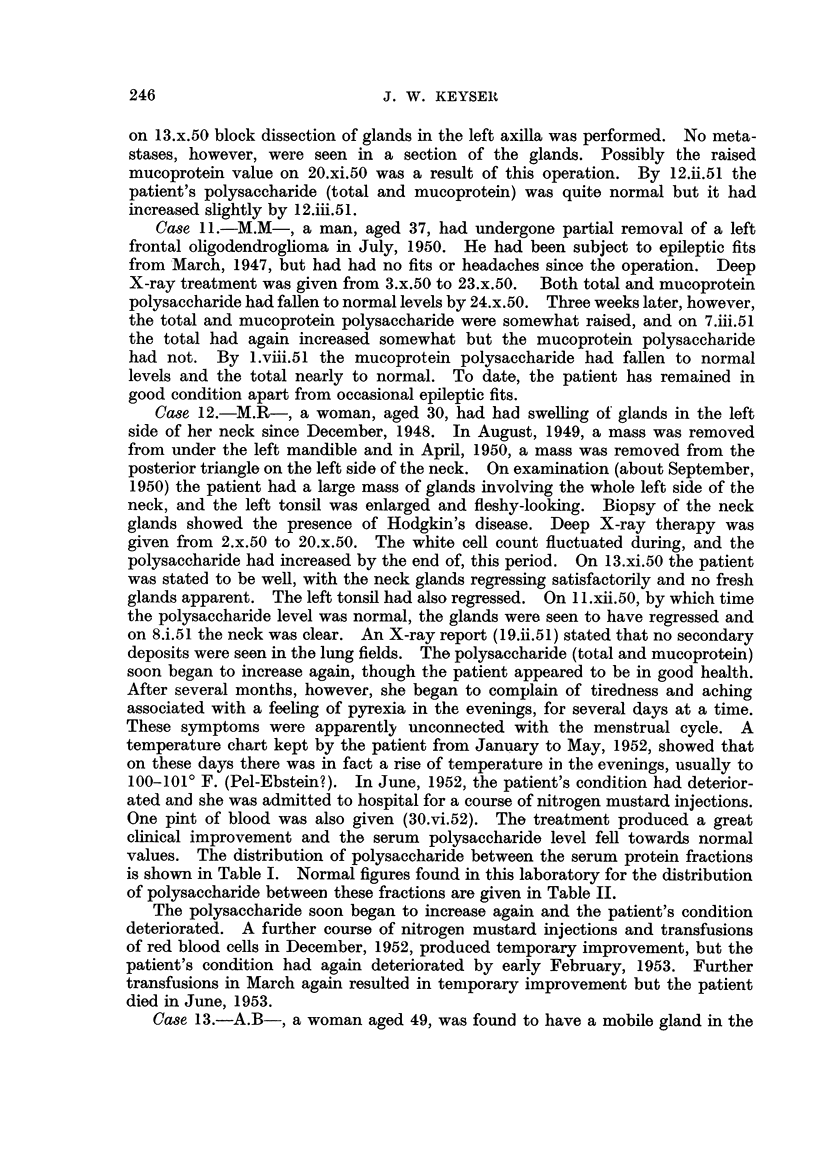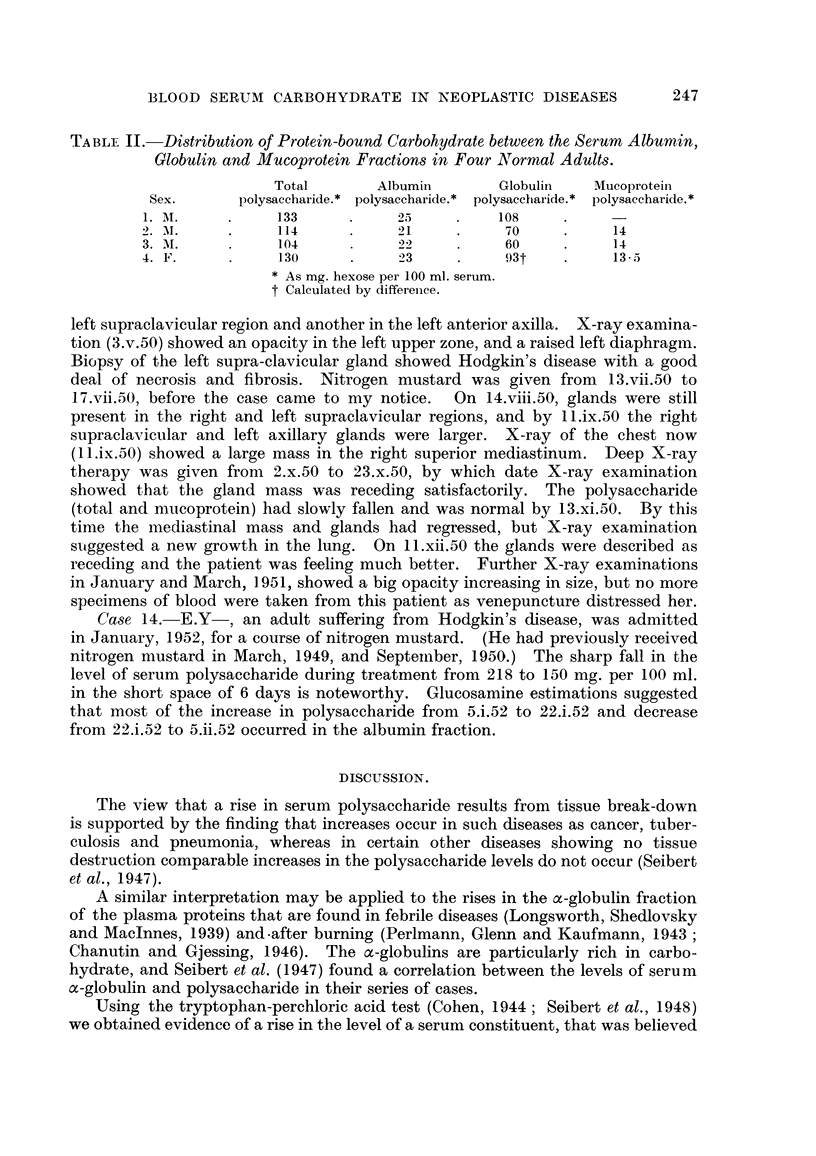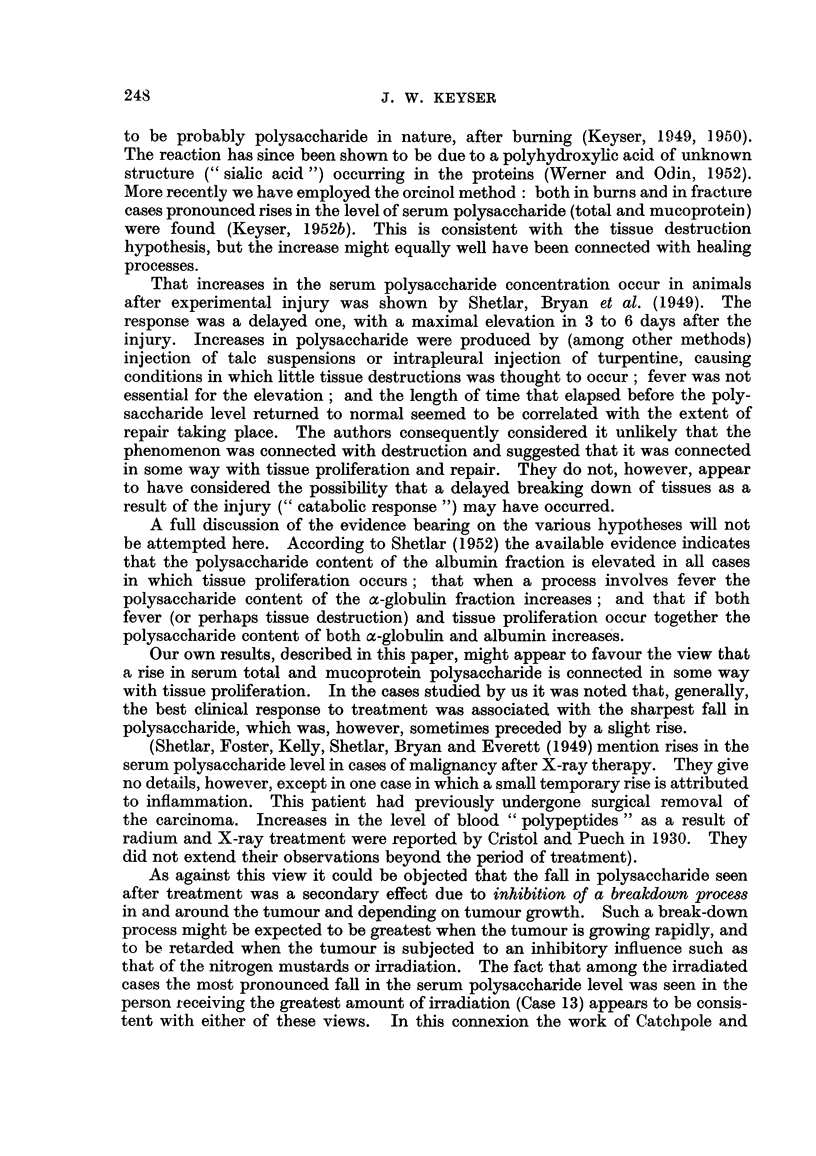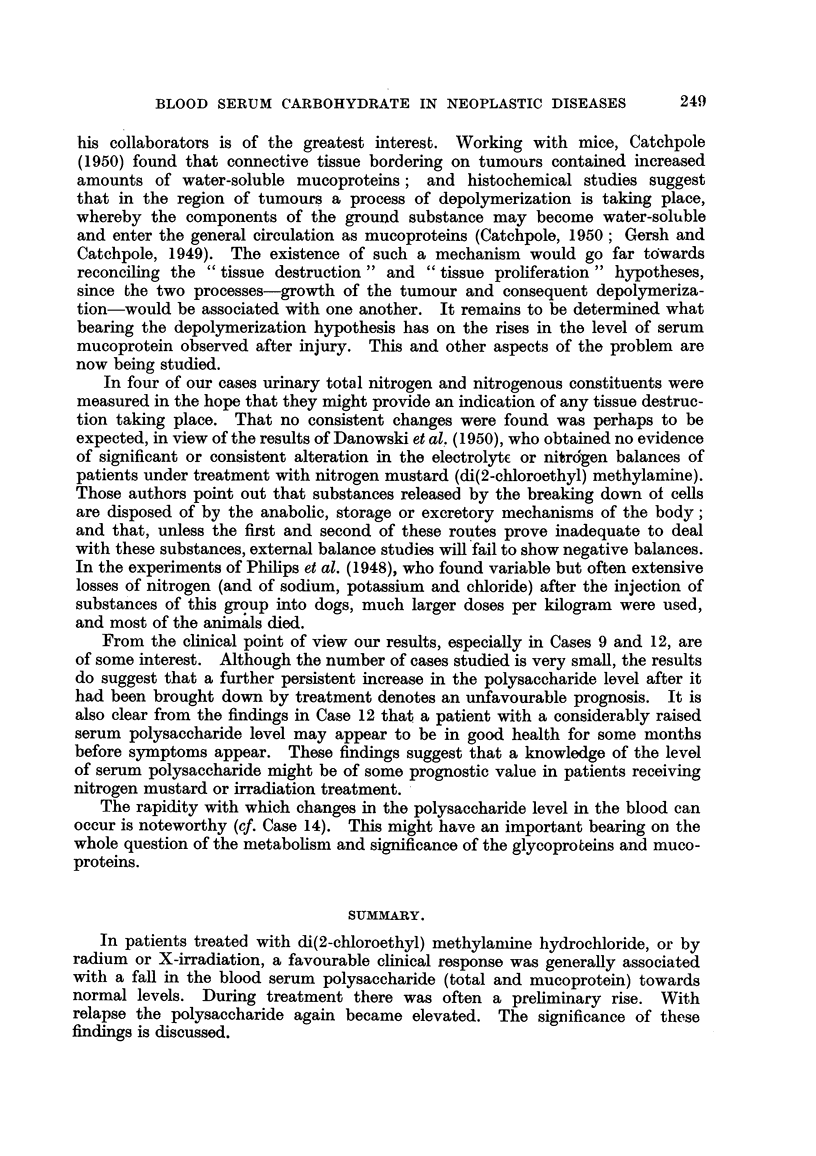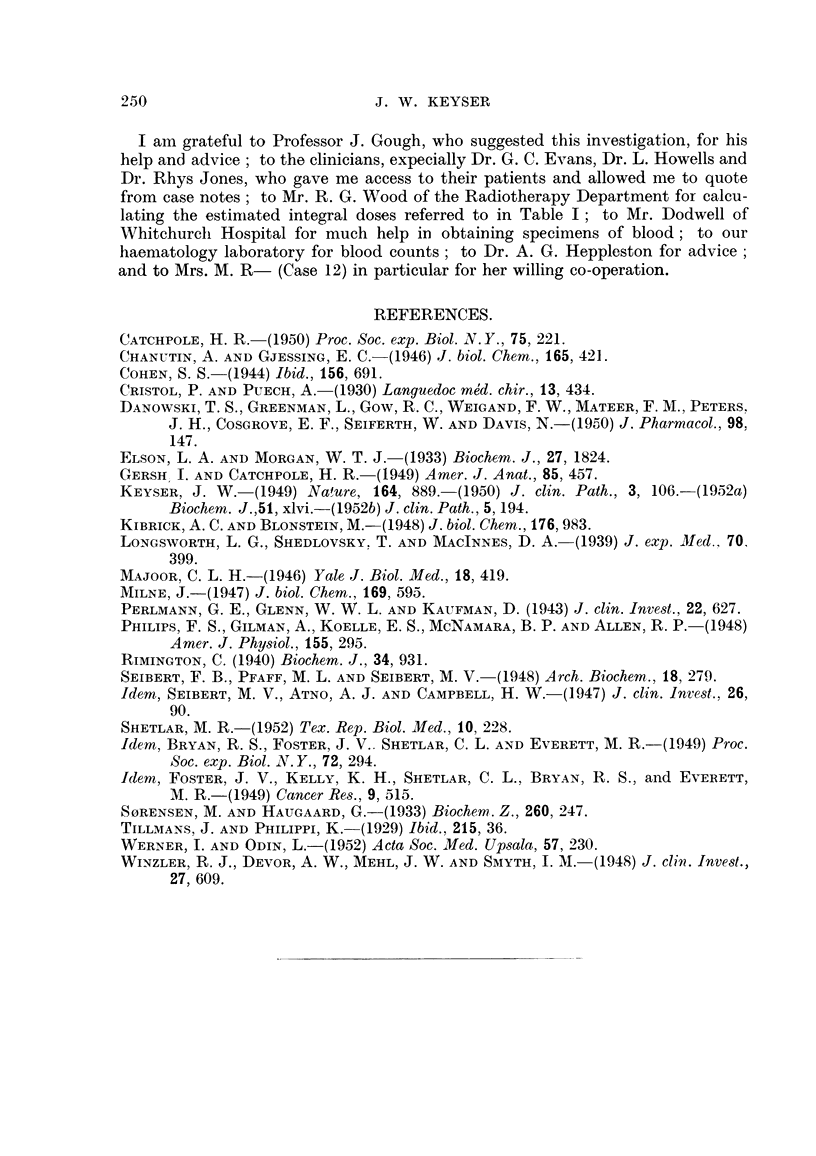# Effect of Nitrogen Mustard or X- or Radium-Irradiation Treatment on the Level of Blood Serum Protein-bound Carbohydrate (Polysaccharide) in Patients with Neoplastic Diseases

**DOI:** 10.1038/bjc.1954.24

**Published:** 1954-06

**Authors:** J. W. Keyser


### EFFECT OF NITROGEN MUSTARD OR X- OR RADIUM-IRRADIATION TREATMENT ON THE LEVEL OF BLOOD SERUM PROTEIN-BOUND CARBOHYDRATE (POLYSACCHARIDE) IN PATIENTS WITH NEOPLASTIC DISEASES.

J. W. KEYSER.

*From the Department of Pathology and Bacteriology, Welsh National School of Medicine, Cardiff.*

Received for publication March 13, 1954.

MANY observations have been published on the effect of disease or injury on the concentration of protein-bound carbohydrate (polysaccharide, protein sugar) or various carbohydrate-rich protein fractions in the blood serum, both in human beings and in animals. A study of the level of serum polysaccharide in various diseases led Seibert, Seibert, Atno and Campbell (1947) to suggest that a raised concentration is associated with tissue destruction. However, Shetlar, Bryan, Foster, Shetlar and Everett (1949) put forward an apparently opposing view, namely, that such increases are a reflection of tissue proliferation or repair rather than of destruction.

This paper deals with an investigation undertaken to find out what effect nitrogen mustard (the bis compound, di(2-chloroethyl) methylamine hydrochloride, was used throughout) or irradiation treatment might have on the serum polysaccharide level in patients with various neoplastic and allied diseases. (In such patients raised values are usually found.) It was hoped that some light might be thrown on any relationship between the polysaccharide level and tissue proliferation or destruction, since the nitrogen mustards and X-irradiation are known to cause tissue destruction or at least to inhibit actively growing tissues. Another object was to find out how much of any increase was contributed by the mucoprotein fraction. A preliminary report of the main findings has been published (Keyser, 1952*a*).

### METHODS.

Fourteen cases were studied, made up of Hodgkin's disease (7), cerebral tumour (1), carcinoma of various types (4), chronic myeloid leucaemia (1) and reticulum cell sarcoma (1).

Blood was obtained by venepuncture immediately before treatment began, then at intervals during and after the course, the serum being separated and kept frozen until the analyses were performed. Total serum protein was separated by alcoholic precipitation (Keyser, 1952*b*). Mucoprotein was separated as described by Winzler, Devor, Mehl and Smyth (1948). Carbohydrate estimations were made by the orcinol method (Tillmans and Philippi, 1929 ; Sørensen and Haugaard, 1933) as described by Rimington (1940). Normal values found in this laboratory by these methods are as follows (Keyser, 1952*b*), expressed as mg.

hexose per 100 ml. serum: total serum protein carbohydrate, 102 to 139 (mean 119, standard deviation 12); mucoprotein carbohydrate, 10·0 to 13·0 (mean 11·9, standard deviation 1·2). In a few cases the albumin polysaccharide was determined after fractionation of the serum proteins with 26 per cent sodium sulphate (Majoor, 1946; Milne, 1947; Kibrick and Blonstein, 1948).

Serum, 1 ml., was incubated at 37° C. with 20 ml. of 27·2 per cent (W/V) sodium sulphate, $Na_2SO_4$, for about 4 hr. The precipitated globulin was filtered off with a 9-cm. filter paper (Whatman No. 5), and the albumin precipitated from 2- to 3-ml. portions of the clear filtrate by the addition of 0·25 volume of approximately 20 per cent perchloric acid and spun down after 10 to 15 minutes. The supernatant fluid was decanted and the tube inverted and allowed to drain. The protein was washed with 2 ml. of 95 per cent (V/V) ethanol and again spun down and the tube drained as before. Finally the protein was dissolved in 0·5 ml. of 0·125 N sodium hydroxide and the hexose estimated by the orcinol method as previously described.

Another portion (4 or 5 ml.) of filtrate was taken for estimation of the albumin.

In one case glucosamine was estimated in the precipitated serum total protein and albumin (26 per cent $Na_2SO_4$ fractionation), by the method of Elson and Morgan (1933) as described by Rimington (1940). Care was taken always to have approximately the same ratio of protein to acid in the hydrolysis from one specimen to another: results should therefore be comparable, though there are known to be numerous sources of error.

The serum tryptophan-perchloric acid reaction (Cohen, 1944; Seibert, Pfaff and Seibert, 1948) was performed on many specimens of serum. In a few cases urine was collected in an attempt to assess the excretion of total nitrogen, creatine, uric acid and amino-nitrogen. Because of the absence of facilities for accurate metabolic work it is possible that not all the urine collections were accurately made; nor was it practicable to carry out nitrogen balances. For these reasons the urine findings are not presented in detail.

### RESULTS.

In general, treatment that resulted in marked clinical improvement and reduction in the size of the tumour or glands was associated with a fall in the level of serum polysaccharide (total and mucoprotein) towards normal values; and with relapse the polysaccharide level again rose.

Sometimes, however, during a course of treatment there was a preliminary rise in the level of polysaccharide before it fell. (It may be that this preliminary rise occurs in all cases. To determine whether this is so it would be necessary to take blood samples at more frequent intervals than we did, but this is not always justifiable.) The tryptophan-perchloric acid reaction paralleled roughly the total serum polysaccharide. No consistent changes in any of the urinary nitrogenous constituents were found during nitrogen mustard treatment (Cases 1, 2, 3 and 14). Creatinuria occurred in Cases 1, 2 and 3 during nitrogen mustard treatment, but not in Case 14.

Table I summarizes our findings. Essential case notes are as follows:

*Case* 1.—W. P—, a man, aged 50, suffering from Hodgkin's disease, was admitted to hospital on 30.iii.50 for his second course of nitrogen mustard. Since the first course of treatment a few months previously there had been an increase in

TABLE I.—*Summary of Findings in Fourteen Cases.*

| Case No. | Age. | Sex. | Diagnosis. | Special treatment (N.M. = nitrogen mustard). | Date. | Serum total protein (g./100 ml.). | Polysaccharide (as mg. galactose-mannose/100 ml.). Total protein. | Polysaccharide (as mg. galactose-mannose/100 ml.). Muco-protein. | Remarks. |
|---|---|---|---|---|---|---|---|---|---|
| 1. | 50 | M. | Hodgkin's disease | N.M. Six 5 - mg. injections 31.iii.50–5.iv.50 | 31.iii.50 | 6·5 | 180 | — | Greatly improved as result of treatment, especially about 3–4 weeks after end of course. |
| | | | | | 3.iv.50 | 6.15 | 160 | — | |
| | | | | | 5.iv.50 | 5·95 | 154 | — | |
| | | | | | 6.iv.50 | 6·25 | 148 | — | |
| | | | | | 6.v.50 | 7·4 | 142 | — | |
| | | | | N.M. Six 5 - mg. injections | 11.vii.50 | 6·35 | 194 | — | Ditto. |
| | | | | | 19.viii.50 | 7·2 | 139 | — | |
| | | | | | 16.ix.50 | 7·05 | 153 | — | |
| | | | | N.M. Four 5-mg., one 6-mg. and one 7-mg. injection 9–14.xii·50 | 8.xii.50 | 6·05 | 188 | 42·5 | Not such good improvement after this course. |
| | | | | | 11.xii.50 | 6·0 | 194 | 50·0 | |
| | | | | | 15.xii.50 | 6·1 | 214 | 54·5 | |
| | | | | | 20.i.51 | 7·2 | 143 | 19·0 | |
| | | | | | 14.iv.51 | 6·45 | 258 | 64·0 | 14.iv.51: Patient appears very ill. Died 12.vii.51. |
| 2. | 36 | F. | Hodgkin's disease | N.M. Six 5 - mg. injections 18.iv.50–23.iv.50 | 18.iv.50 | 6·9 | 231 | — | |
| | | | | | 20.iv.50 | 6·7 | 235 | — | |
| | | | | | 25.iv.50 | 6·15 | 230 | — | Discharged 27.iv.50 for convalescence. |
| | | | | | 11.v.50 | 7·7 | 189 | — | |
| | | | | | 28.viii.50 | 7·5 | 253 | — | Re-admitted 24.viii.50 (relapse). |
| 3. | 59 | M. | Hodgkin's disease and pleural effusion | Aspiration of chest, 8.iv.50 and 14.iv.50 | 19.iv.50 | 5·35 | 160 | — | |
| | | | | | 20.iv.50 | 5·6 | 169 | — | |
| | | | | | 22.iv.50 | 5·6 | 170 | — | |
| | | | | N.M. Six 5 - mg. injections 20.iv.50–25.iv.50 | 25.iv.50 | 5·9 | 174 | — | |
| | | | | | 9.v.50 | 6·0 | 159 | — | Discharged 17.v.50, oedema and chest signs reduced. Died 28.v.50. |
| 4. | 26 | M. | Chronic myeloid leukaemia | N.M. Six 5 - mg. injections 30.vi.50–5.vii.50 | 30.vi.50 | 6·5 | 145 | — | Improved on treatment. |
| | | | | | 6.vii.50 | 7·15 | 151 | — | |
| | | | | | 10.viii.50 | 7·6 | 118 | — | |
| | | | | | 1.ix.50 | 7·65 | 144 | — | Relapse. |

| Case No. | Age. | Sex. | Diagnosis. | Special treatment. (N.M. = nitrogen mustard.) | Date. | Serum total protein (g./100 ml.). | Polysaccharide (as mg. galactose-mannose/100 ml.) Total protein. | Muco-protein. | Remarks. |
|---|---|---|---|---|---|---|---|---|---|
| 5. | 76 | M. | Reticulum cell sarcoma | N.M. Six 5 - mg. injections 10.ii.51–15.ii.51 | 6.ii.51 | 6·5 | 180 | 28·0 | |
| | | | | | 10.ii.51 | 6·55 | 198 | — | |
| | | | | | 14.ii.51 | 7·15 | 211 | — | |
| | | | | | 15.ii.51 | 7·0 | 204 | 31·0 | |
| | | | | | 19.ii.51 | 6·15 | 174 | 27·0 | Discharged 23.ii.51. |
| | | | | | 21.iii.51 | 6·85 | 240 | 46·0 | Later admitted to hospital, and died 27.iii.51. |
| 6. | 58 | M. | Hodgkin's disease | N.M. 5 mg. on 28.ii.51 and 1, 2, 5 and 7.iii.51 | 8.viii.50 | 5·9 | 180 | — | |
| | | | | | 28.ii.51 | 4·45 | 166 | 58·0 | |
| | | | | | 1.iii.51 | 4·25 | 174 | — | |
| | | | | | 3.iii.51 | 4·0 | 182 | 56·0 | |
| | | | | | 8.iii.51 | 4·0 | 186 | 58·0 | |
| | | | | | 10.iii.51 | 4·25 | 204 | 60·0 | Died 14.iii.51. |

| Case No. | Age. | Sex. | Diagnosis. | Special treatment. (M.S.D. = maximum skin dose. G.D. = given dose.) | Date. | Serum total protein (g./100 ml.). | Polysaccharide (as mg. galactose-mannose/100 ml.) Total protein. | Muco-protein. | Remarks. |
|---|---|---|---|---|---|---|---|---|---|
| 7. | 71 | M. | Squamous carcinoma of dorsum of left hand | Radium mould. Dose 5500 r. 79·4hr. in 8 days (21–28.ix.50). Estimated integral dose 8 M.gm. r. (very approximate, chiefly effect of the Ra on the whole body) | 20.ix.50 | 7·05 | 148 | 25·0 | |
| | | | | | 28.ix.50 | 7·25 | 148 | 21·0 | |
| | | | | | 20.xi.50 | — | 155 | 22·5 | 20.xi.50: Healing. |
| | | | | | 18.xii.50 | 7·15 | 146 | 24·5 | 18.xii.50: Healed. Covered with scar. Died 9.iv.51 (myocardial degeneration). |
| 8. | 39 | F. | Carcinoma of left breast | Deep X-rays (palliative). 220 kv. H.V.L. = 1·5 mm. Cu. Dose: M.S.D. 3000 r. G.D. 3000 r. (each field). Time: 16 exposures in 10 days (20–29.ix.50). Estimated integral dose 7 M.gm. r. | 20.ix.50 | 6·75 | 203 | 65·0 | |
| | | | | | 29.ix.50 | 6·6 | 239 | 64·0 | |
| | | | | | 29.xi.50 | 7·45 | 148 | 41·5 | 29.xi.50: Feeling better. Breast healing, but left axilla and left supraclavicular fossa may need treatment. Died: 22.iii.51. |

TABLE I.—*Summary of Findings in Fourteen Cases—cont.*

| Case No. | Age. | Sex. | Diagnosis. | Special treatment. (G.D. = given dose.) | Date. | Serum total (g./protein 100 ml.). | Polysaccharide (as mg. galactose-mannose/100 ml.) Total protein. | Polysaccharide (as mg. galactose-mannose/100 ml.) Muco-protein. | Remarks. |
|---|---|---|---|---|---|---|---|---|---|
| 9. | 69 | M. | Carcinoma of right upper alveolus involving cheek, palate, mouth | Deep X-rays (palliative). 220 kv. H.V.L. = 1·5 mm. Cu. G.D. 3296 r., right face. G.D. 2496 r. left face. Time: 16 exposures in 10 days (21–30.ix.50). Estimated integral dose 3 M.gm. r. | 20.ix.50 | 6·55 | 212 | 37·0 | Good response to treatment, but died 12.ii.51 (metastases in cervical glands). |
| | | | | | 30.ix.50 | 7·0 | 181 | 30·0 | |
| | | | | | 15.xi.50 | 6·85 | 165 | 21·5 | |
| | | | | | 13.xii.50 | — | — | 43·5 | |
| 10. | 45 | M. | Carcinoma of skin of left forearm (? Pneumoconiosis.) | Radium mould. Dose 6000 r. Time 73 hr in 8 days (28.ix–5.x.50). Estimated integral dose 8 M.gm. r. | 26.ix.50 | 7·3 | 149 | 14·0 | 13.x.50: Block dissection of glands in left axilla. |
| | | | | | 6.x.50 | 7·45 | 148 | — | |
| | | | | | 20.xi.50 | 7·05 | 143 | 18·5 | 20.xi.50: Still has large area at primary site which is slow to heal. |
| | | | | | 12.ii.51 | 7·15 | 123 | 11·0 | 15.i.51: Primary slowly healing. |
| | | | | | 12.iii.51 | 7·65 | 138 | 18·0 | 12.iii.51: Small raised area at lower part of primary. No glands. |
| | | | | | 16.vii.51 | 7·45 | 156 | 10·5 | |
| 11. | 37 | M. | Left frontal cerebral tumour | Deep X-rays (palliative). 250 kv. H.V.L. = 1·5 mm. Cu. Dose: T.D. 4000 r., G.D. 2700 r. (each field). Time: 45 exposures in 21 days (3–23.x.50). Estimated integral dose 4 M.gm. r. | 26.ix.50 | 7·05 | 169 | 31·0 | |
| | | | | | 24.x.50 | 6·75 | 118 | 14·0 | |
| | | | | | 15.xi.50 | 6·75 | 147 | 19·5 | |
| | | | | | 7.iii.51 | 7·2 | 164 | 17·0 | |
| | | | | | 1.viii.51 | 7·25 | 150 | 13·0 | |
| 12. | 30 | F. | Hodgkin's disease | Deep X-rays (radical). 250 kv. H.V.L. = 1·5 mm. Cu. Dose: T.D. 3500 r. G.D. 2925 r. (right and left neck). Time: 30 exposures in 19 days (2–20.x.50). Estimated integral dose 9 M.gm. r. | 27.ix.50 | 6·95 | 160 | 22·0 | |
| | | | | | 21.x.50 | 7·00 | 170 | 24·0 | 13.xi.50: Well. Neck glands regressing well. No fresh glands. |
| | | | | | 13.xi.50 | 6·25 | 149 | 16·0 | |
| | | | | | 11.xii.50 | 7·0 | 124 | 9·5 | |
| | | | | | 7.iii.51 | 7·1 | 158 | 16·0 | 2.iv.51: Left tonsil still prominent. |
| | | | | | 30.vi.51 | 6·95 | 198 | 22·5 | |
| | | | | | 3.xii.51 | 6·9 | 196 | 30·0 | |
| | | | | | 14.i.52 | 6·45 | 176 | 22·0 | |
| | | | | | 10.iii.52 | 6·8 | 184 | 28·0 | |

| | | | | | | | | | |
|---|---|---|---|---|---|---|---|---|---|
| | | | | | 21.iv.52 | 6·3 | 216 | 38·5 | |
| | | | | | 21.vi.52 | 6·0 | 218(61)* | 26·5 | 21.vi.52 : Serum albumin 3·1 G./100 ml. |
| | | | | 23.vi.52 : 4 mg. N.M. | 24.vi.52 | 6·0 | 228(61)* | 31·5 | 24.vi.52 : Serum albumin 3·2 |
| | | | | 24.vi.52 : 4 ,, ,, | 26.vi.52 | 6·0 | 230(64)* | 36·5 | 26.vi.52 : Serum albumin 3·0 |
| | | | | 25.vi.52 : 5 ,, ,, | | | | | |
| | | | | 26.vi.52 : 6 ,, ,, | 28.vi.52 | 6·05 | 230(64)* | 32·0 | 28.vi.52 : Serum albumin 3·1 5.vii.52 Serum albumin 4·0 |
| | | | | 30.vi.52 : 1 pint blood | 5.vii.52 | 6·65 | 196(60)* | 27·5 | 21.vii.52 : Serum albumin 4·5 |
| | | | | | 21.vii.52 | 6·45 | 152(46)* | 19·5 | 21.vii.52 : Looks well. No glands palpable. |
| | | | | | 22.ix.52 | 7·0 | 220(46)* | 43·5 | |
| | | | | | 2.ii.53 | 5·9 | 214(46)* | 43·5 | 22.ix.52 : Serum albumin 3·1 G./100 ml. 2.ii.53 : Serum albumin 3·1 June 1953 : Died. |
| 13. | 49 | F. | Hodgkin's disease | Deep X-rays. Radical: 250 kv. Dose: T.D. 3000 r., G.D. 3006 r. Anterior chest. Posterior chest. Time: 36 exposures in 21 days (2–23. x.50). Estimated integral dose 20 M. gm. r. | 27.ix.50 | 6·45 | 247 | 78·0 | |
| | | | | | 24.x.50 | 6·3 | 155 | 29·0 | 23.x.50 : Gland mass receding satisfactorily. |
| | | | | | 13.xi.50 | 6·7 | 136 | 12·0 | |
| 14. | — | M. | Hodgkin's disease | N.M. Six 5-mg. doses, 21–25.i.52 | 5.i.52 | 7·0 | 183 | 27·0 | |
| | | | | | 22.i.52 | 6·5 | 218 | 34·0 | |
| | | | | | 24.i.52 | 6·45 | 202 | 37.0 | |
| | | | | | 26.i.52 | 6·2 | 175 | 32·0 | |
| | | | | | 28.i.52 | 5·9 | 150 | 25·0 | |
| | | | | | 5.ii.52 | 7·0 | 142 | 22·0 | |
| | | | | | 16.ii.52 | — | 130 | 13·0 | |
| | | | | | 5.iv.52 | 6·5 | 122 | 19·0 | |
| | | | | | 1.vi.52 | 6·1 | 144 | 20·0 | |
| | | | | | 30.viii.52 | 6·5 | 168 | 21·5 | |
| | | | | | 7.iii.52 | 6·5 | 171 | 27·0 | |

* Figures in parentheses represent the polysaccharide in the albumin fraction the size of the glands. Daily injections resulted in improvement corresponding with a fall in the total white cell count and total polysaccharide, which was down nearly to normal a week after the beginning of treatment (see Table I). About the middle of June the patient began to complain of weakness and tiredness and noticed that the glands in his neck were once more increasing in size. He was re-admitted on 10.vii.50 and again nitrogen mustard produced clinical improvement and a fall in both white cell count and total polysaccharide. After another relapse he was given a fourth course of nitrogen mustard from 9.xii.50 to 14.xii.50. The patient was discharged, a little improved, on 18.xii.50. This time there was an increase in the polysaccharide (total and mucoprotein) during treatment but by a few weeks after the end of the injections it had again fallen nearly to normal levels. Unfortunately serial white cell counts were not made during the last period. The patient was last seen on 14.iv.51, when he appeared quite ill and his total and mucoprotein polysaccharides were very high. He died on 12.vii.51.

*Case* 2.—E.W—, a woman, aged 36, with Hodgkin's disease, was admitted to hospital on 18.iv.50 for a course of nitrogen mustard therapy. She had previously had four such courses. During treatment there was a slight rise in the serum total polysaccharide, then a fall. A leucocytosis apparently also occurred during the first few days but the white cell count fell markedly during the latter part of the course. Pyrexia persisted during treatment. No significant fall in the polysaccharide level occurred during the course of injections, but within 3 weeks after the end of the course it had fallen by 40 mg. per 100 ml. The patient was discharged to a convalescence home on 27.iv.50 and then home on 12.v.50. She felt very well until about the end of July, but she was again admitted in the following month with marked enlargement of the lymph glands of the neck and enlargement of the axillary glands. Her serum polysaccharide on admission was again very high. Unfortunately it proved exceedingly difficult to obtain further specimens of blood from this patient, on account of venous thrombosis, so that no further systematic study of the case was possible. She was later admitted to another hospital for radiotherapy and died there.

*Case* 3.—O.K—, a man, aged 59, with Hodgkin's disease and a left pleural effusion, was admitted on 4.iv.50 for nitrogen mustard treatment. After aspiration of the chest and six daily injections of nitrogen mustard the oedema of his arms improved considerably and " chest signs " improved. He was pyrexial during most of the course of treatment. On 17.v.50 he was discharged for convalescence, but he died eleven days later. There was a raised polysaccharide level on admission and no significant change during or after treatment.

*Case* 4.—A.W—, a man, aged 26, had chronic myeloid leucaemia. He was admitted to hospital for a course of nitrogen mustard and was subsequently discharged (" improved "). The serum polysaccharide fell after treatment, but was again slightly raised on readmission in September, when the patient was thin and pale and showed evidence of loss of weight. There were firm, mobile, moderately enlarged glands palpable in his neck, axillae and groins.

No further biochemical studies were made in this case.

*Case* 5.—G.R—, a man, aged 76, had noticed the appearance of large masses in the right side of his neck over the previous 18 months with a rapid increase in size in the 6 weeks preceding his admission to hospital. There were also lumps under his axillae and in the groins. A biopsy of a lymph gland on 3.ii.51 showed reticulum cell sarcoma. A course of six daily injections of nitrogen mustard was followed by a slight reduction in the size of the lymph nodes and the patient was discharged (" improved ") on 23.ii.51. During the nitrogen mustard treatment his serum polysaccharide (total and mucoprotein) rose somewhat, and it then fell but did not reach normal. The white cell count fell during treatment. Soon after discharge he was admitted to another hospital. His serum total and mucoprotein polysaccharide were found to be very high, and he died on 27.iii.51.

*Case* 6.—F.W—, aged 58, had previously received nitrogen mustard treatment for Hodgkin's disease, and on admission showed multiple skin nodules. He was given injections of nitrogen mustard. As shown in Table I the total serum polysaccharide increased steadily, but the mucoprotein polysaccharide showed only slight changes. The patient died on 14.iii.51. *Post mortem*, liver, spleen and mediastinal glands showed diffuse involvement.

*Case* 7.—T.C—, a man, aged 71, had had a wart on the back of his hand for several years. During the 8 months immediately before admission it had increased in size (on admission it measured 5 × 3 × 0·75 cm.) and for several weeks it had been ulcerating. A biopsy showed squamous carcinoma. The patient was treated with radium from 21.ix.50 to 28.ix.50. There were only slight changes in the polysaccharide, which was only slightly above normal on 20.xi.50, when the hand was stated to be healing. By February, 1951, it was completely healed, but the patient was taken ill about January and died on 9.iv.51 (myocardial degeneration).

*Case* 8.—V.C—, a woman, aged 39, had noticed a swelling on her left breast 20 years previously. It had been ulcerating for 4 years. On examination it was found that the whole of the left breast was involved by growth, and biopsy showed squamous carcinoma. There was no radiological evidence of secondary deposits in chest, thorax or pelvis but there was body destruction of the radicles and laminae of vertebra L3. At the end of deep X-ray therapy the total polysaccharide had increased by 36 mg. per 100 ml. but the mucoprotein fraction was about the same. At the end of 2 months, however, the total polysaccharide had fallen considerably and the mucoprotein fraction to a lesser extent. Healing of the medial half of the right breast area was progressing satisfactorily. The patient died a few months later. The cause of death was stated to be carcinoma of the left breast.

*Case* 9.—D.W—, a man, aged 69, had an extensive papillary squamous carcinoma which probably originated in the posterior part of a right upper alveolus but had spread medially to involve part of the soft palate and laterally to fill the cheek alveolus sulcus and was extending down on the posterior part of the right cheek. The size of the tumour was estimated at about 4 cm. in diameter in all axes. No glands were felt in the patient's neck. Biopsy of the palate revealed squamous carcinoma. The clinical response to treatment with deep X-rays was good. By the end of treatment the polysaccharide (total and mucoprotein) had fallen considerably and 6 weeks later it was still lower. On 13.xii.50, however, the mucoprotein polysaccharide was found to have increased sharply, and the patient died on 12.ii.51 (carcinoma of alveolus, metastases in cervical glands).

*Case* 10.—P.W—, a man, aged 45, had noticed a pimple developing on the dorsal side of the upper part of the forearm. On examination the growth was 23 sq. cm. in area. A section showed that it was a squamous cell neoplasm, probably malignant. Radium treatment was given from 28.ix.50 to 5.x.50, and on 13.x.50 block dissection of glands in the left axilla was performed. No metastases, however, were seen in a section of the glands. Possibly the raised mucoprotein value on 20.xi.50 was a result of this operation. By 12.ii.51 the patient's polysaccharide (total and mucoprotein) was quite normal but it had increased slightly by 12.iii.51.

*Case* 11.—M.M—, a man, aged 37, had undergone partial removal of a left frontal oligodendroglioma in July, 1950. He had been subject to epileptic fits from March, 1947, but had had no fits or headaches since the operation. Deep X-ray treatment was given from 3.x.50 to 23.x.50. Both total and mucoprotein polysaccharide had fallen to normal levels by 24.x.50. Three weeks later, however, the total and mucoprotein polysaccharide were somewhat raised, and on 7.iii.51 the total had again increased somewhat but the mucoprotein polysaccharide had not. By 1.viii.51 the mucoprotein polysaccharide had fallen to normal levels and the total nearly to normal. To date, the patient has remained in good condition apart from occasional epileptic fits.

*Case* 12.—M.R—, a woman, aged 30, had had swelling of glands in the left side of her neck since December, 1948. In August, 1949, a mass was removed from under the left mandible and in April, 1950, a mass was removed from the posterior triangle on the left side of the neck. On examination (about September, 1950) the patient had a large mass of glands involving the whole left side of the neck, and the left tonsil was enlarged and fleshy-looking. Biopsy of the neck glands showed the presence of Hodgkin's disease. Deep X-ray therapy was given from 2.x.50 to 20.x.50. The white cell count fluctuated during, and the polysaccharide had increased by the end of, this period. On 13.xi.50 the patient was stated to be well, with the neck glands regressing satisfactorily and no fresh glands apparent. The left tonsil had also regressed. On 11.xii.50, by which time the polysaccharide level was normal, the glands were seen to have regressed and on 8.i.51 the neck was clear. An X-ray report (19.ii.51) stated that no secondary deposits were seen in the lung fields. The polysaccharide (total and mucoprotein) soon began to increase again, though the patient appeared to be in good health. After several months, however, she began to complain of tiredness and aching associated with a feeling of pyrexia in the evenings, for several days at a time. These symptoms were apparently unconnected with the menstrual cycle. A temperature chart kept by the patient from January to May, 1952, showed that on these days there was in fact a rise of temperature in the evenings, usually to 100–101° F. (Pel-Ebstein?). In June, 1952, the patient's condition had deteriorated and she was admitted to hospital for a course of nitrogen mustard injections. One pint of blood was also given (30.vi.52). The treatment produced a great clinical improvement and the serum polysaccharide level fell towards normal values. The distribution of polysaccharide between the serum protein fractions is shown in Table I. Normal figures found in this laboratory for the distribution of polysaccharide between these fractions are given in Table II.

The polysaccharide soon began to increase again and the patient's condition deteriorated. A further course of nitrogen mustard injections and transfusions of red blood cells in December, 1952, produced temporary improvement, but the patient's condition had again deteriorated by early February, 1953. Further transfusions in March again resulted in temporary improvement but the patient died in June, 1953.

*Case* 13.—A.B—, a woman aged 49, was found to have a mobile gland in the

TABLE II.—*Distribution of Protein-bound Carbohydrate between the Serum Albumin, Globulin and Mucoprotein Fractions in Four Normal Adults.*

| Sex. | Total polysaccharide.* | Albumin polysaccharide.* | Globulin polysaccharide.* | Mucoprotein polysaccharide.* |
|---|---|---|---|---|
| 1. M. | 133 | 25 | 108 | — |
| 2. M. | 114 | 21 | 70 | 14 |
| 3. M. | 104 | 22 | 60 | 14 |
| 4. F. | 130 | 23 | 93† | 13·5 |

* As mg. hexose per 100 ml. serum.

† Calculated by difference.

left supraclavicular region and another in the left anterior axilla. X-ray examination (3.v.50) showed an opacity in the left upper zone, and a raised left diaphragm. Biopsy of the left supra-clavicular gland showed Hodgkin's disease with a good deal of necrosis and fibrosis. Nitrogen mustard was given from 13.vii.50 to 17.vii.50, before the case came to my notice. On 14.viii.50, glands were still present in the right and left supraclavicular regions, and by 11.ix.50 the right supraclavicular and left axillary glands were larger. X-ray of the chest now (11.ix.50) showed a large mass in the right superior mediastinum. Deep X-ray therapy was given from 2.x.50 to 23.x.50, by which date X-ray examination showed that the gland mass was receding satisfactorily. The polysaccharide (total and mucoprotein) had slowly fallen and was normal by 13.xi.50. By this time the mediastinal mass and glands had regressed, but X-ray examination suggested a new growth in the lung. On 11.xii.50 the glands were described as receding and the patient was feeling much better. Further X-ray examinations in January and March, 1951, showed a big opacity increasing in size, but no more specimens of blood were taken from this patient as venepuncture distressed her.

*Case* 14.—E.Y—, an adult suffering from Hodgkin's disease, was admitted in January, 1952, for a course of nitrogen mustard. (He had previously received nitrogen mustard in March, 1949, and September, 1950.) The sharp fall in the level of serum polysaccharide during treatment from 218 to 150 mg. per 100 ml. in the short space of 6 days is noteworthy. Glucosamine estimations suggested that most of the increase in polysaccharide from 5.i.52 to 22.i.52 and decrease from 22.i.52 to 5.ii.52 occurred in the albumin fraction.

### DISCUSSION.

The view that a rise in serum polysaccharide results from tissue break-down is supported by the finding that increases occur in such diseases as cancer, tuberculosis and pneumonia, whereas in certain other diseases showing no tissue destruction comparable increases in the polysaccharide levels do not occur (Seibert *et al.*, 1947).

A similar interpretation may be applied to the rises in the $\alpha$-globulin fraction of the plasma proteins that are found in febrile diseases (Longsworth, Shedlovsky and MacInnes, 1939) and after burning (Perlmann, Glenn and Kaufmann, 1943; Chanutin and Gjessing, 1946). The $\alpha$-globulins are particularly rich in carbohydrate, and Seibert *et al.* (1947) found a correlation between the levels of serum $\alpha$-globulin and polysaccharide in their series of cases.

Using the tryptophan-perchloric acid test (Cohen, 1944; Seibert *et al.*, 1948) we obtained evidence of a rise in the level of a serum constituent, that was believed to be probably polysaccharide in nature, after burning (Keyser, 1949, 1950). The reaction has since been shown to be due to a polyhydroxylic acid of unknown structure (" sialic acid ") occurring in the proteins (Werner and Odin, 1952). More recently we have employed the orcinol method : both in burns and in fracture cases pronounced rises in the level of serum polysaccharide (total and mucoprotein) were found (Keyser, 1952*b*). This is consistent with the tissue destruction hypothesis, but the increase might equally well have been connected with healing processes.

That increases in the serum polysaccharide concentration occur in animals after experimental injury was shown by Shetlar, Bryan *et al.* (1949). The response was a delayed one, with a maximal elevation in 3 to 6 days after the injury. Increases in polysaccharide were produced by (among other methods) injection of talc suspensions or intrapleural injection of turpentine, causing conditions in which little tissue destructions was thought to occur ; fever was not essential for the elevation ; and the length of time that elapsed before the polysaccharide level returned to normal seemed to be correlated with the extent of repair taking place. The authors consequently considered it unlikely that the phenomenon was connected with destruction and suggested that it was connected in some way with tissue proliferation and repair. They do not, however, appear to have considered the possibility that a delayed breaking down of tissues as a result of the injury (" catabolic response ") may have occurred.

A full discussion of the evidence bearing on the various hypotheses will not be attempted here. According to Shetlar (1952) the available evidence indicates that the polysaccharide content of the albumin fraction is elevated in all cases in which tissue proliferation occurs ; that when a process involves fever the polysaccharide content of the $\alpha$-globulin fraction increases ; and that if both fever (or perhaps tissue destruction) and tissue proliferation occur together the polysaccharide content of both $\alpha$-globulin and albumin increases.

Our own results, described in this paper, might appear to favour the view that a rise in serum total and mucoprotein polysaccharide is connected in some way with tissue proliferation. In the cases studied by us it was noted that, generally, the best clinical response to treatment was associated with the sharpest fall in polysaccharide, which was, however, sometimes preceded by a slight rise.

(Shetlar, Foster, Kelly, Shetlar, Bryan and Everett (1949) mention rises in the serum polysaccharide level in cases of malignancy after X-ray therapy. They give no details, however, except in one case in which a small temporary rise is attributed to inflammation. This patient had previously undergone surgical removal of the carcinoma. Increases in the level of blood " polypeptides " as a result of radium and X-ray treatment were reported by Cristol and Puech in 1930. They did not extend their observations beyond the period of treatment).

As against this view it could be objected that the fall in polysaccharide seen after treatment was a secondary effect due to *inhibition of a breakdown process* in and around the tumour and depending on tumour growth. Such a break-down process might be expected to be greatest when the tumour is growing rapidly, and to be retarded when the tumour is subjected to an inhibitory influence such as that of the nitrogen mustards or irradiation. The fact that among the irradiated cases the most pronounced fall in the serum polysaccharide level was seen in the person receiving the greatest amount of irradiation (Case 13) appears to be consistent with either of these views. In this connexion the work of Catchpole and his collaborators is of the greatest interest. Working with mice, Catchpole (1950) found that connective tissue bordering on tumours contained increased amounts of water-soluble mucoproteins; and histochemical studies suggest that in the region of tumours a process of depolymerization is taking place, whereby the components of the ground substance may become water-soluble and enter the general circulation as mucoproteins (Catchpole, 1950; Gersh and Catchpole, 1949). The existence of such a mechanism would go far towards reconciling the "tissue destruction" and "tissue proliferation" hypotheses, since the two processes—growth of the tumour and consequent depolymerization—would be associated with one another. It remains to be determined what bearing the depolymerization hypothesis has on the rises in the level of serum mucoprotein observed after injury. This and other aspects of the problem are now being studied.

In four of our cases urinary total nitrogen and nitrogenous constituents were measured in the hope that they might provide an indication of any tissue destruction taking place. That no consistent changes were found was perhaps to be expected, in view of the results of Danowski *et al.* (1950), who obtained no evidence of significant or consistent alteration in the electrolyte or nitrogen balances of patients under treatment with nitrogen mustard (di(2-chloroethyl) methylamine). Those authors point out that substances released by the breaking down of cells are disposed of by the anabolic, storage or excretory mechanisms of the body; and that, unless the first and second of these routes prove inadequate to deal with these substances, external balance studies will fail to show negative balances. In the experiments of Philips *et al.* (1948), who found variable but often extensive losses of nitrogen (and of sodium, potassium and chloride) after the injection of substances of this group into dogs, much larger doses per kilogram were used, and most of the animals died.

From the clinical point of view our results, especially in Cases 9 and 12, are of some interest. Although the number of cases studied is very small, the results do suggest that a further persistent increase in the polysaccharide level after it had been brought down by treatment denotes an unfavourable prognosis. It is also clear from the findings in Case 12 that a patient with a considerably raised serum polysaccharide level may appear to be in good health for some months before symptoms appear. These findings suggest that a knowledge of the level of serum polysaccharide might be of some prognostic value in patients receiving nitrogen mustard or irradiation treatment.

The rapidity with which changes in the polysaccharide level in the blood can occur is noteworthy (*cf.* Case 14). This might have an important bearing on the whole question of the metabolism and significance of the glycoproteins and mucoproteins.

### SUMMARY.

In patients treated with di(2-chloroethyl) methylamine hydrochloride, or by radium or X-irradiation, a favourable clinical response was generally associated with a fall in the blood serum polysaccharide (total and mucoprotein) towards normal levels. During treatment there was often a preliminary rise. With relapse the polysaccharide again became elevated. The significance of these findings is discussed.

I am grateful to Professor J. Gough, who suggested this investigation, for his help and advice ; to the clinicians, expecially Dr. G. C. Evans, Dr. L. Howells and Dr. Rhys Jones, who gave me access to their patients and allowed me to quote from case notes ; to Mr. R. G. Wood of the Radiotherapy Department for calculating the estimated integral doses referred to in Table I ; to Mr. Dodwell of Whitchurch Hospital for much help in obtaining specimens of blood ; to our haematology laboratory for blood counts ; to Dr. A. G. Heppleston for advice ; and to Mrs. M. R— (Case 12) in particular for her willing co-operation.

### REFERENCES.

CATCHPOLE, H. R.—(1950) *Proc. Soc. exp. Biol. N.Y.*, **75**, 221.
CHANUTIN, A. AND GJESSING, E. C.—(1946) *J. biol. Chem.*, **165**, 421.
COHEN, S. S.—(1944) *Ibid.*, **156**, 691.
CRISTOL, P. AND PUECH, A.—(1930) *Languedoc méd. chir.*, **13**, 434.
DANOWSKI, T. S., GREENMAN, L., GOW, R. C., WEIGAND, F. W., MATEER, F. M., PETERS, J. H., COSGROVE, E. F., SEIFERTH, W. AND DAVIS, N.—(1950) *J. Pharmacol.*, **98**, 147.
ELSON, L. A. AND MORGAN, W. T. J.—(1933) *Biochem. J.*, **27**, 1824.
GERSH, I. AND CATCHPOLE, H. R.—(1949) *Amer. J. Anat.*, **85**, 457.
KEYSER, J. W.—(1949) *Nature*, **164**, 889.—(1950) *J. clin. Path.*, **3**, 106.—(1952*a*) *Biochem. J.*,**51**, xlvi.—(1952*b*) *J. clin. Path.*, **5**, 194.
KIBRICK, A. C. AND BLONSTEIN, M.—(1948) *J. biol. Chem.*, **176**, 983.
LONGSWORTH, L. G., SHEDLOVSKY, T. AND MACINNES, D. A.—(1939) *J. exp. Med.*, **70**, 399.
MAJOOR, C. L. H.—(1946) *Yale J. Biol. Med.*, **18**, 419.
MILNE, J.—(1947) *J. biol. Chem.*, **169**, 595.
PERLMANN, G. E., GLENN, W. W. L. AND KAUFMAN, D. (1943) *J. clin. Invest.*, **22**, 627.
PHILIPS, F. S., GILMAN, A., KOELLE, E. S., MCNAMARA, B. P. AND ALLEN, R. P.—(1948) *Amer. J. Physiol.*, **155**, 295.
RIMINGTON, C. (1940) *Biochem. J.*, **34**, 931.
SEIBERT, F. B., PFAFF, M. L. AND SEIBERT, M. V.—(1948) *Arch. Biochem.*, **18**, 279.
*Idem*, SEIBERT, M. V., ATNO, A. J. AND CAMPBELL, H. W.—(1947) *J. clin. Invest.*, **26**, 90.
SHETLAR, M. R.—(1952) *Tex. Rep. Biol. Med.*, **10**, 228.
*Idem*, BRYAN, R. S., FOSTER, J. V., SHETLAR, C. L. AND EVERETT, M. R.—(1949) *Proc. Soc. exp. Biol. N.Y.*, **72**, 294.
*Idem*, FOSTER, J. V., KELLY, K. H., SHETLAR, C. L., BRYAN, R. S., and EVERETT, M. R.—(1949) *Cancer Res.*, **9**, 515.
SØRENSEN, M. AND HAUGAARD, G.—(1933) *Biochem. Z.*, **260**, 247.
TILLMANS, J. AND PHILIPPI, K.—(1929) *Ibid.*, **215**, 36.
WERNER, I. AND ODIN, L.—(1952) *Acta Soc. Med. Upsala*, **57**, 230.
WINZLER, R. J., DEVOR, A. W., MEHL, J. W. AND SMYTH, I. M.—(1948) *J. clin. Invest.*, **27**, 609.